# Interventions to reduce the stigma of mental health at work: a narrative review

**DOI:** 10.1186/s41155-023-00255-1

**Published:** 2023-05-22

**Authors:** Raúl Ramírez-Vielma, Pamela Vaccari, Félix Cova, Sandra Saldivia, Alexis Vielma-Aguilera, Pamela Grandón

**Affiliations:** 1 Departamento de Psicología, Facultad de Ciencias Sociales, Universidad de Concepción, Barrio Universitario S/N, Concepción, Chile; 2 Departamento de Psiquiatría y Salud Mental, Facultad de Medicina, Universidad de Concepción, Concepción, Chile

**Keywords:** Social stigma, Workplace, Interventions to reduce stigma, Mental health

## Abstract

**Background:**

While there are reviews of the literature on mental health stigma reduction programs, very few have focused on the workplace. Objective: We sought to identify, describe and compare the main characteristics of the interventions to reduce the stigma towards mental health at work.

**Method:**

The search of original articles (2007 to 2022) was carried out in the Web of Science Core Collection and Scopus databases, selecting 25 articles from the key terms: 1. Stigma, 2. Workplace, 3. Anti-stigma intervention/program, 4. Mental health. Results: These interventions can be effective in changing the knowledge, attitudes, and behaviors of workers towards people with mental health problems, although further verification of these results is needed as they are limited to date.

**Discussion and conclusion:**

Interventions to reduce stigma in the workplace could create more supportive work environments by reducing negative attitudes and discrimination and improving awareness of mental disorders.

## Background

### Stigma towards people with mental health problems

Stigma and discrimination related to mental health are global and multifaceted problems. The impact of experienced and anticipated discrimination is severe, impacting different aspects, such as poor access to health services, reduced life expectancy, exclusion from higher education and employment, and victimization, among others (Clement et al., 2015; Fox et al., 2018; Lawrence & Kisely, 2010; Sharac et al., 2010). For most people, these consequences are worse than the experience of the mental disorder itself (Gronholm et al., 2017). In addition, stigma and discrimination can lead to social isolation, low self-esteem, reluctance to seek treatment, and social rejection (Aakre et al., 2015; Corrigan et al., 2011; Knifton et al., 2009). These consequences are interrelated, increasing mental health difficulties for individuals who may experience high-stress levels while living with the constant threat of being stigmatized (Link & Phelan, 2006). It is estimated that stigmatizing attitudes and behaviors are present in 40% to 70% of people in Latin America (Mascayano et al., 2016).

As defined initially by Goffman (1963), stigma is a profoundly discrediting and isolating process of differentiation, othering, and discrimination toward a person who receives a socially devalued label. In the case of people who stigmatize, it can be understood as a combination of problems of knowledge (ignorance), attitudes (prejudice), and behavior (discrimination) (Thornicroft et al., 2008).

Stigma is a multi-component construct that involves processes of labeling, stereotyping, prejudice, social exclusion, loss of status, and discrimination, all within a context of differential power between the stigmatized and stigmatized groups (Link & Phelan, 2001). Stigma is also far-reaching and can be proximal to the person experiencing a problem, such as prejudice in intimate relationships, or it can take a more distal form, such as structural discriminatory laws and practices that exist in organizations and governments (Bos et al., 2013; Cook et al., 2014; Thornicroft, 2006).

Stigma is experienced in various contexts, including the workplace, and includes public and self-stigma elements (Malachowski & Kirsch, 2013).

### Actions to reduce stigma towards people with mental health problems

Considering its effects, stigma reduction appears to be an imperative and priority public health need that requires mitigation. According to Gronholm et al. (2017), public health programs to reduce discrimination and stigma should be based on a series of decisions: the scope of mental disorders to include, explicitly or implicitly; the level of intervention, whether structural, interpersonal, or self-stigma; the group to intervene, whole population versus choosing target groups, and if opting for the latter, which groups are priority targets in terms of frequency and/or severity of the mental health problem; what focus an intervention should have for a given group and level; and how to evaluate impact.

Strategies to reduce stigma have been categorized in terms of education (replacing myths about mental disorders with accurate knowledge), contact (using direct or indirect interactions with people who have a diagnosis of mental disorder), and protest (organized groups demand changes in stigmatizing attitudes and representations of mental disorder) (Corrigan & O'Shaughnessy, 2007; Stuart et al., 2012).

Contact as education is a widely used intervention strategy (Gronholm et al., 2017; Knifton et al., 2009); however, the most successful programs are multi-component; although they employ educational strategies, they are not limited to them but favor daily contact with people with severe mental disorders and, some, also consider the development of social interaction skills (Knaak et al., 2014; Thornicroft et al., 2016). On the other hand, leading approaches to reduce self-stigma use interventions that increase coping skills, self-esteem, empowerment, hope, subjective perception of recovery, and help-seeking behavior in those affected (Alonso et al., 2019; Mittal et al., 2012). Stangl et al. (2013) have suggested that these strategies address multiple domains and levels of stigma, from an ecological model, to enrich the perspective of analysis on interventions.

One context of intervention to reduce stigma is the workplace, which, in line with what the ecological model posits, implies recognizing that for its reduction, work must be done at the individual levels -microsystem- as well as the group and interactive context -mesosystem-. Following this line of argument, Stuart (2004) highlights as a strategic guideline for addressing stigma the identification of the employment and workplace experiences of people with mental health problems to describe the extent and nature of stigma, as well as the social and organizational characteristics (such as policies, procedures, management structures or programs) that promote or prevent its occurrence in the workplace.

The impact of weak mental health at work is widely recognized and involves work losses associated with absenteeism, presenteeism, and turnover, with a high economic cost to organizational systems (Czabała et al., 2011; Hanisch et al., 2016). For the same reason and given the high prevalence of mental health problems in both the general and working population, the workplace is increasingly being recognized as an important target for mental health promotion, prevention, and intervention (Malachowski & Kirsh, 2013), something that implies necessarily considering interventions with workers.

Unfortunately, research on the effectiveness of interventions to reduce stigma at work is limited; mainly, educational strategies and direct contact with people who have experienced a mental disorder are used, and some of these have been found to reduce stigma, although the evidence presented is not entirely conclusive (Corbière et al., 2009; Malachowski & Kirsh, 2013; Szeto & Dobson, 2010). Because of this, there is a need to understand better the strategies and their effectiveness in combating stigma in the workplace (Corrigan & Fong, 2014).

### Relevance of interventions to reduce stigma in the workplace

Szeto and Dobson (2010) provide two arguments for the importance of studying interventions to reduce mental health stigma in the workplace. The first is the low awareness rate of stigma in these contexts, considering that awareness or recognition does not necessarily equate to understanding or comprehension. Interventions could complement broader efforts to reduce stereotypes, prejudice, and discrimination toward those experiencing a mental disorder by increasing awareness and providing more information on a sustained basis. A second argument comes from research in Social Psychology; a subject's appraisal varies as a function of context (Barden et al., 2004; Rudman & Lee, 2002). The literature suggests that the knowledge and information learned as part of public anti-stigma campaigns are related to the context in which they were learned. Positive, counter-stereotypical representations of those experiencing a mental disorder may not be activated in the workplace, as this was not the context in which this information was acquired (Szeto & Dobson, 2010).

In the same vein, Gawronski et al. (2010) have suggested that counter-attitudinal information delivered as part of an intervention should be given in multiple contexts, thus eliminating the contextual dependence on this information. Similarly, Krupa et al. (2009) have argued that stigma processes are sensitive to and derived from the context and the social relationships embedded within them. These authors have also found that assumptions made in the literature about general stigma may not be operative within the work context.

Thus, the development and implementation of effective stigma reduction programs specifically designed for the workplace are of great importance. While public efforts to reduce stigma have yielded mixed results (Corrigan & Shapiro, 2010; Szeto & Dobson, 2010), developing strategies tailored to particular contexts, such as the workplace, may be a more promising route to stigma reduction. On the other hand, participation in stigma reduction programs, for example, in human resource development, could be mandatory in an organizational setting, making it more time- and information-intensive (Hanisch et al., 2016).

This review is justified by some of the arguments presented, particularly the importance of the context in which stigma arises and can be changed, the limited knowledge of stigma and its nature in the workplace, as well as the author’s recognition of the promising feasibility of stigma-reducing interventions tailored to specific contexts such as work and organizations.

### The current study

Based on the above background, an important challenge still pending is to determine the characteristics of interventions aimed at reducing the stigma towards mental health problems in the work context. Therefore, this review seeks to identify, describe and compare the primary interventions to reduce mental health stigma in the workplace, at the micro and meso level. It is also expected to detect some research gaps and provide some recommendations for improvement. We searched for articles between 2007 and 2022 and the geographical scope of these articles was only conditioned by the search languages English and Spanish. The aim of the study fits a narrative review model as we aim to approach a broad range of issues within a given topic (Onwuegbuzie & Frels, 2016), which in this case are interventions to reduce mental health stigma at work. Furthermore, our study corresponds to the narrative review subcategory "general literature review" which provides a review of the most important and critical aspects of current knowledge on the topic (Onwuegbuzie & Frels, 2016).

## Method

**Fig. 1:**
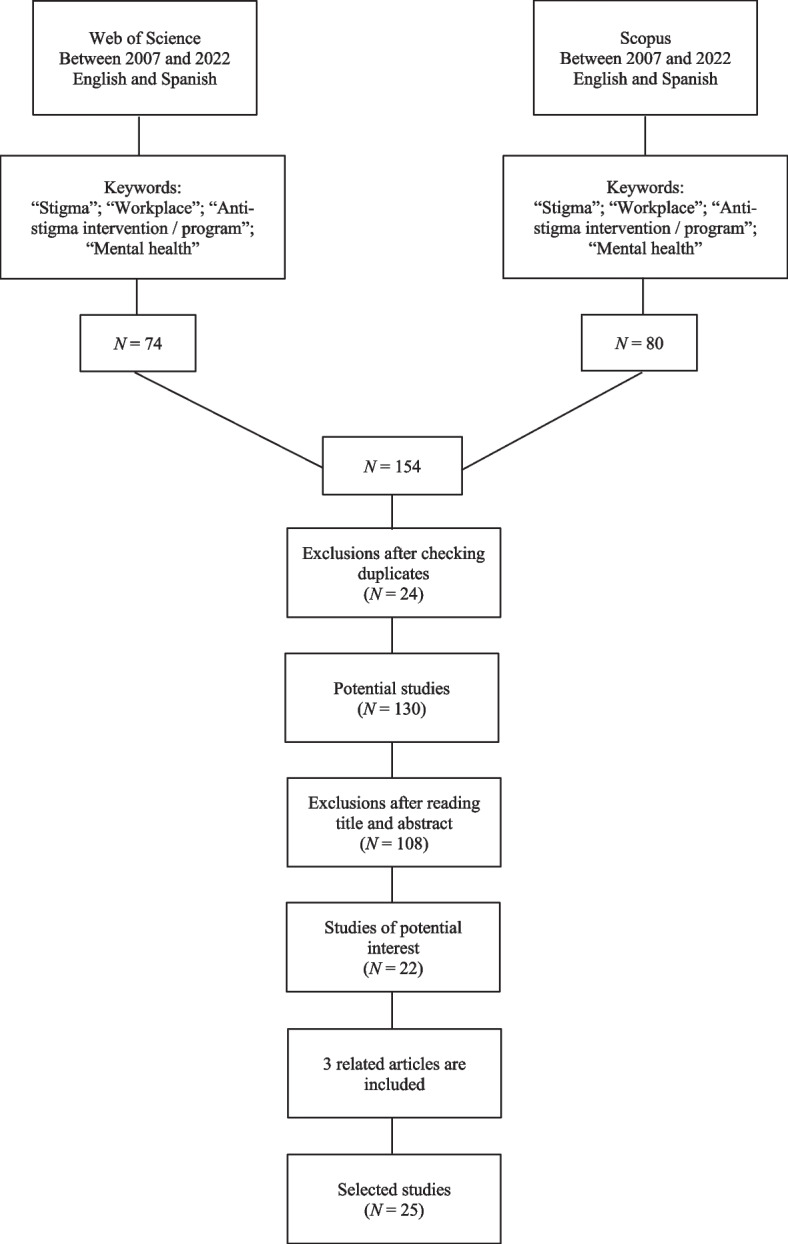
Bibliographic search flow diagram

A narrative review of the literature was conducted, which is defined as a “written document that critically reviews the relevant literature on a research topic, presenting a logical case that establishes a thesis delineating what is currently known about the subject” (Machi & McEvoy, 2022, p. 1). Corresponding to the subcategory "general literature review", this narrative review was conducted following the quality guidelines established by Baethge et al. (2019). The search for original articles from January 2007 to August 2022 was carried out in the Web of Science Core Collection and Scopus databases. It was decided to work with these databases because they ensure a wide variety of articles of scientific impact. The approximate starting period of the search was defined by reference to Szeto and Dobson (2010), who formulated one of the first reviews on the topic with an emphasis on the pioneering Mental Health First Aid Programme (2007) as a joint international effort to reduce stigma towards mental health problems including the workplace context. As stated above, the geographical scope of the search was only conditioned by the search languages, which were English and Spanish. The key terms used in the title, abstract, and keywords sections were: 1.- Stigma; 2.- Workplace; 3.- Anti-stigma intervention/program; 4.- Mental health. These terms were combined with logical functions and operators using the “OR, AND & NOT”, specific for each search engine, to reduce and specify the resulting articles. Thus, 154 articles were found in both databases, 74 in Web of Science Core Collection and 80 in Scopus. By discarding duplicates, this number was reduced to 130, and 22 articles were selected for this narrative review. Three articles that met the inclusion criteria were added and cited in the studies by Malachowski and Kirsh (2013) and Hanisch et al. (2016). Figure 1 is the flow chart of the search performed.

The following process was carried out to select the studies: reading the title and abstract, reading the full text, and selecting the articles to be included. Regarding the inclusion criteria, the main parameter followed was the type of interventions to reduce mental health stigma in workers, specifically the selection of empirical studies referring to workplace interventions to reduce mental health stigma in workers. Regarding exclusion criteria, case studies, dissertations, letters to the editor, and duplicate records were eliminated from the analysis. All these steps were followed to safeguard the quality of the literature search for the narrative review (Baethge et al., 2019). Finally, articles in a language other than English or Spanish were not considered.

Twenty-five empirical studies were included, which were characterized and are presented in the following section (Dimoff et al., 2016; Dobson et al., 2021; Gould et al., 2007; Griffiths et al., 2016; Hamann et al., 2016; Hanisch et al., 2017; Hossain et al., 2009; Jensen et al., 2016; Jorm et al., 2010; Knifton et al., 2009; Krameddine et al., 2013; Kubo et al., 2018; Lunasco et al., 2010; Moffitt et al., 2014; Moll et al., 2015; Moll et al., 2018a, 2018b; Moll et al., 2018a, 2018b; Nishiuchi et al., 2007; Oakie et al., 2018; Quinn et al., 2011; Reavley et al., 2018; Shann et al., 2019; Svensson & Hansson, 2014; Szeto et al., 2019; Tynan et al., 2018). The analysis plan was underpinned by selecting, reading, synthesizing, and exposing these studies on interventions to reduce mental health stigma in the workplace.

## Results

From the selected articles, it was possible to establish a series of categories of analysis that allowed the characterization of the interventions. These categories are 1) Scope of application; 2) Design proposal and intervention modality; 3) Objective of the intervention; 4) Impact of the intervention. The analysis of each dimension is presented below; the specific results are included in Table 1.

**Table 1:** Characterization of interventions to reduce mental health stigma in the workplace

Authors of the studies	Scope of application			Design proposal and intervention modality		Objective of the intervention	Impact of the intervention	
	Country	Participants	Target population	Design of the intervention	Components of the intervention		Key findings	Limitations
Dimoff et al. (2016)	Canada	Managers and supervisors in universities and telecommunications companies (*N* = 420)	Public and private sector Higher education and telecommunications	Randomized controlled clinical trial design	Intervention with a session length of 3 h. The training program is configured for the mental health literacy of organizational leaders, with a sequence of early identification and recognition, early engagement or action, and assessment, planning, and monitoring	Seeks to increase the mental health literacy of organizational leaders in terms of improving knowledge and attitudes towards mental health and fostering self-efficacy and intention to promote mental health at work	The intervention had a direct effect on knowledge and self-efficacy but indirect effects on attitudes and intentions. Also, the program led to a reduction in the duration of short-term disability claims	Relatively small sample size in both studies. The results, are based only on data from two organizations and there is a need to replicate these findings in a variety of organizational contexts
Dobson et al. (2021)	Canada	Office, kitchen, and health area maintenance staff (*N* = 123)	Public sector	Cluster randomized design, with pretest, post-test, and 3-month follow-up in 2 implementation groups in 4 workplaces	The intervention has a duration of 3 months. This is the Working Mind Program designed to reduce mental health stigmatization in the workplace	It seeks to reduce stigmatizing attitudes towards mental disorders, improve resilience and promote mental health in the workplace	Qualitative data provided additional evidence of program benefits in participants. The program effectively decreased mental health stigma and increased self-reported resilience and coping skills at pre and post-assessment in both groups. The effects of the program were sustained through the 3-month follow-up	The cluster randomization yielded groups that were not equivalent at baseline. The moderate attrition at the presurvey time point in the delayed group and at the time of follow-up assessment in the immediate group. Future program evaluations should consider longer follow-up intervals
Gould et al. (2007)	United Kingdom	Active military personnel (*N* = 124)	Public sector Military context in the UK Royal Navy	Quasi-experimental pre-and post-test design	The intervention is called TRiM, that is, Trauma Risk Management, and it is a psychoeducational management strategy (based on peer groups). It is based on a didactic and role-playing strategy. Intervention with a duration of 2.5 days	It seeks to modify negative attitudes towards people with PTSD and stress and increase supportive behaviors in the face of possible risk situations	It improved attitudes towards people with post-traumatic stress and seeking support from trained personnel. However, there was no significant effect on changing attitudes toward seeking help from normal support networks in the military and general health	The generalizability is a major limitation of the study because the military is a unique organization. A further limitation is that this was a brief longitudinal study. Self-report questionnaires were used and it is possible that initial positive attitudes occurred because of participants providing socially desirable responses
Griffiths et al. (2016)	Australia	Officials of a multidepartmental government organization (*N* = 507)	Public sector Government organizations	Randomized controlled clinical trial design, with baseline, post-intervention, and six-month follow-up outcome measures	It is a two-module online induction program (MH-Guru) on mental health at work, focusing on depression and anxiety. It uses a simple, interactive multimedia format that contains graphics and exercises in the program. The intervention lasts two weeks	It seeks to increase anxiety and depression literacy, decrease negative attitudes to these conditions, provide counseling to supervisors and colleagues to help co-workers with mental health problems, and promote help-seeking	A brief online educational program effectively reduced stigma and improved mental health literacy among the staff of different seniority positions and work settings. In turn, the program was well accepted by the trainees	The study attrition at 6-months. Other limitations were the failure to separate out information and treatment help seeking outcomes and the absence of a measure of treatment help-seeking behaviour for the 6-month period prior to the final follow-up. The design involved randomization at the individual rather than departmental level. The study it focused on one type of organization
Hamann et al. (2016)	Germany	Managers or members of the human resources department (*N* = 580) from different organizations	Private sector The business environment in the HR area of several companies focuses on the management level	Pre-experimental design (Pre- and post-test design)	Workshop modality, highly standardized and manualized workshop format, integrating didactic lectures, small group activities (role-playing), and showing the experience of workers with depression. Intervention duration of 1 or 1.5-day seminar	Seeks to reduce stigma towards people with depression by bosses and managers	Stigma towards depression was significantly reduced, and managers' knowledge of mental disorders and how they manifest themselves at work was improved	Participants may not be representative for managers. The design was pre-post and therefore lack a control group. Short-term results were obtained and cannot predict whether the effects observed will last for longer or translate into practice
Hanisch et al. (2017)	United Kingdom	Managers or directors of a multinational company (*N* = 48)	Private sector Multinational business environment	Pre-experimental design (pre-post-test design with a 3-month follow-up for training evaluation)	Leadership Training in Mental Health Promotion (LMHP) is a digital game-based training program for leaders. The training is a single session, lasting 1.5 to 2 h	Seeks to promote employees' mental health and reduce the stigma regarding mental problems at work	A positive impact was found on mental health knowledge, attitudes toward people with mental health problems, and self-efficacy to deal with mental health situations; the exception was the intention to promote employees' mental health, which was initially already high	The study lacked a control group due to formal restrictions of the participating site. To measure knowledge, a quiz was developed which was not validated. Participants might have been presensitized as a result of stigma reduction efforts that have been going on in the UK
Hossain et al. (2009)	Australia	Extension agents and advisors (EAAs) who have frequent contact with farmers may manifest mental health problems (*N* = 32)	Private sector Agricultural activity sector	Pre-experimental design (Pre- and post-test design)	The MHFA, or Mental Health First Aid, was applied as a knowledge and skills training modality. This intervention promotes the recognition of mental health risk behaviors in users. There is no report of session duration	It seeks to increase the skills and knowledge of agents in recognizing symptoms of mental disorders to provide initial help and offer guidance	An improvement in the ability to recognize a mental disorder was obtained, as well as an increase in the participants' confidence to help people with mental health problems and a decrease in social distance. Finally, positive beliefs about treatment increased	The design was pre-post and therefore lack a control group. This study has limitations in its ability to identify the use and impact of training. It is recommended further studies be carried out to evaluate the impact of training on the mental health and wellbeing
Jensen et al. (2016)	Denmark	Workers from various organizations (*N* = 566)	Private, public, and NGO sectors Public, private, and non-governmental organizations (NGOs)	Randomized controlled clinical trial design, with a waiting list control group	The MHFA or Mental Health First Aid was applied. The intervention was based on exercises, knowledge presentations, and discussions. The course lasted two days with 12 h of training	Seeks to improve confidence in helping people with a mental disorder, enhancing the knowledge and ability to recognize mental disorders, and increasing positive attitudes towards these people	A significant difference was found between trained employees in the intervention group compared to the control group at a 6-month follow-up on elements of confidence in contacting, talking to, and providing help to people suffering from mental health problems. Participants improved in knowledge and ability to recognize schizophrenia. However, changes in attitudes were limited	The higher attrition rate among participants in the intervention group compared to the control group, a tendency too in other studies of the effect of MHFA training. The trial also implied a risk of contamination between the two groups (e.g., workplace cross-groups). The limited focus on helping-behaviour in the questionnaire
Jorm et al. (2010)	South Africa	School teachers (*N* = 423)	Public and private sector School context	Randomized controlled clinical trial design	The intervention is based on the MHFA or Mental Health First Aid. The first part focused on mental health knowledge, and the second part was on skills development. One or two-day session, 7 h long	It seeks to increase knowledge on depression, suicide, and anxiety disorders, decrease stigmatizing attitudes and increase confidence in providing help to those who present these problems	Mental health knowledge increased, beliefs about treatment changed, some aspects of stigma were reduced, and confidence in providing help to students and colleagues increased. Much of the changes found were maintained six months after the training	This effectiveness trial carried out under real-life rather than optimal conditions. The pre-test assessment had to be carried out after group assignment. Two schools withdrew from the project because changed circumstances did not allow them to conduct the training as planned
Knifton et al. (2009)	United Kingdom	Workers in contact with people experiencing mental health problems (*N* = 137)	It is not clear whether the organizations belong to the private or public sector Benefits, housing, employment, voluntary sector agencies	Pre-experimental design (Pre- and post-test design)	Workshop modality employs service user narratives, experiential group learning, and didactic teaching approaches. The workshop duration was 6 h	It seeks to promote positive attitudes, challenging negative stereotypes towards mental health problems and generating a positive behavioral intention in the target audience	There was a significant improvement in participants' knowledge of mental health problems. Prejudices regarding unpredictability and recovery in people with mental disorders were significantly modified, but dangerousness did not change. Social distance significantly improved only in relation to "moderate" social contact	There was no control group, so quantitative results cannot be compared with a non-intervention group. The sample size within this study was quite modest (137, 63 post-data) and in particular had a smaller number of males
Krameddine et al. (2013)	Canada	Police officers (*N* = 663)	Public sector Police and law enforcement providers	Pre-experimental design (Pre- and post-test design)	Trained using carefully protocolized role-play with actors in 6 real situations. Intervention of 1-day duration	It seeks to improve interactions with those with a mental disorder by increasing empathy, communication skills, and ability to handle potentially difficult situations	There were changes in attitudes toward people with mental disorders and significant improvements in both directly and indirectly, measured behaviors	The design was pre-post and therefore lack a control group. Anonymous self-report measures of attitudes and supervisor surveys were used, and no interviews were conducted
Kubo et al. (2018)	Japan	Workers in a manufacturing company (*N* = 91)	Private sector Industrial manufacturing sector	Pre-experimental design (Pre- and post-test design)	It consists of a training program based on MHFA or Mental Health First Aid, following the basic principles of this international intervention. The program is 12 h long, 2 h each day	It seeks to generate changes in confidence and practical skills to support early depression and prevent suicide, as well as to reduce stigma towards mental health problems	The program had a positive impact on promoting support for co-workers with mental health problems	The design was pre-post and therefore lack a control group. Evaluation using self-rated questionnaire and short-term follow up period were other limitations of the study
Lunasco et al. (2010)	United States	Military (*N* = 320)	Public sector Military. The training was done in military operations	Pre-experimental design (Pre- and post-test design)	Uses the One Shot—One Kill (OS-OK), a culturally sensitive mental health prevention program. Intervention duration in a 2-day, 4-h format	It seeks to reduce stigma and improve help-seeking behaviors in a "culture of war" context	Positive responses were observed, demonstrating that the intervention program is culturally sound, increasing help-seeking among military members	The design was pre-post and therefore lack a control group. Although data were collected regarding soldiers’ perceptions of their mastery of skills across health-related areas, this data was not included in the study
Moffitt et al. (2014)	United Kingdom	Firefighters (*N* = 176)	Public sector Public protection services	Randomized controlled clinical trial design	Fire department line managers were randomly assigned to 3 training conditions: LWW (a program aimed at promoting wellness and awareness of mental health problems), MHFA (Mental Health First Aid, a protocolized international training program), or LS (a purely educational control condition). Duration LWW and MHFA 2 days and LS 1 h	Seeks to promote wellness and reduce mental health stigma	LWW and MHFA courses were associated with statistically significant improvements in attitudes toward mental disorders and knowledge/self-efficacy around mental health	The quantitative evaluation is limited, as one of the questionnaires had untested psychometric properties. The control condition was limited as it was only offered for one hour, making comparison with two-day training problematic. It may have been possible to conduct a more in-depth qualitative analysis with a smaller number of participants
Moll et al. (2015)	Canada	Health officials (*N* = 200)	Public sector Health organizations	Randomized controlled clinical trial design	Two intervention modalities were compared: Beyond Silence, a peer-led program tailored to the healthcare workplace, and Mental Health First Aid (MHFA), a standardized literacy-based training program. Both interventions had a duration of 12 h of training	It seeks to increase mental health literacy, reduce stigmatized beliefs, and increase help-seeking behaviors or the helpfulness of healthcare employees toward people with mental disorders	Changes in knowledge, attitudes toward mental disorders, and help-seeking and help-giving behavior were observed, although the study will continue, as this was an initial report	Although the research questions are analyzed through a clinical trial, a mixed methods approach can be particularly important for understanding program implementation. The results cannot be generalized on a larger scale to other workplaces
Moll et al., (2018a, 2018b)	Canada	Health officials (*N* = 192)	Public sector. Health organizations	Randomized controlled clinical trial design	Two intervention modalities were compared: Beyond Silence, a contact-based workplace education program, and Mental Health First Aid (MHFA), a standard mental health literacy training program. Both interventions had a duration of 12 h of training	Seeks to promote mental health literacy to promote early intervention and support for health officials with mental health problems	Neither program led to significant increases in help-seeking or disclosure behaviors. Both programs increased mental health literacy, improved attitudes toward seeking treatment, and decreased stigmatizing beliefs, with more sustained changes in Beyond Silence with respect to the most prominent stigmatizing beliefs	The study was conducted with hospital employees in one geographic region of Ontario. Participants in the study were volunteers, so they may be more open and receptive to the educational programs. Research is needed to explore whether the findings are replicated in smaller organizations in rural settings in other jurisdictions
Moll et al., (2018a, 2018b)	Canada	Health officials (*N* = 182)	Public sector Health organizations	Randomized controlled clinical trial design	The interventions Beyond Silence, a contact-based on-the-job training program, and Mental Health First Aid (MHFA), a standard mental health literacy program, were compared. Both interventions had a duration of 12 h of training	Seeks to promote mental health education in the workplace for health care workers	Five common key strengths of the programs were: contact-based educational approach, information tailored to the work context, varied stakeholder perspectives, sufficient time to integrate and apply to learn, and organizational support. Beyond Silence outperforms the first three	Generalizability is limited by the small sample of participants who were likely the early adopters and motivated to participate in workplace mental health training. The study was restricted to a 1 medium and 1 large hospital in the same urban center
Nishiuchi et al. (2007)	Japan	Supervisors of a traditional sake beer brewer (*N* = 46)	Private sector Sake brewery industrial area	Randomized controlled clinical trial design	The education program includes guidelines for promoting worker mental health in the intervention group. In terms of duration, it was a single-session intervention	Seeks to determine whether a stress reduction education program influences supervisors' knowledge, attitudes (stigma), and behavior for stress management	The supervisor's knowledge and behavior in managing stress in the workplace were improved for at least six months. Stigmatizing attitudes were also reduced	Lack of the process evaluation is a limitation. The study was conducted within a single workplace, and therefore our study population was not sufficiently representative of all supervisors across occupations. A six- month follow-up alone cannot determine the educational effect
Oakie et al. (2018)	Canada	Workers from the same organization (*N* = 40)	Sector not identified Unidentified organizational scope	Quasi-experimental pre-and post-test design	The Peer Health Awareness Training (CHAT) program was implemented, complementary to the Mental Health Awareness Training (MHAT) program. CHAT is based on providing information about mental health and promoting awareness through coping techniques. The duration of the intervention was 2 h	It seeks to influence variables such as knowledge, stigma, self-efficacy to recognize and address mental health problems, intention to promote good mental health, and willingness to use resources	Employees trained with CHAT showed increased knowledge, self-efficacy, mental health promotion, and willingness to use resources. However, this was not replicated in the case of stigma reduction, where there were no significant differences	The small sample size was a significant challenge and reduced the statistical power of the analyses. All of the measures used were self-report. Another limitation is the lesser control of the quasi-experimental design
Quinn et al. (2011)	United Kingdom	Employees of public and private organizations (*N* = 87)	Private and public sectors Construction and telecommunications associations sector	Pre-experimental design (Pre- and post-test design)	Characterized by service user narratives, group experiential learning, and didactic teaching approaches. Intervention of 9 sessions of one day each	It seeks to promote positive attitudes and challenge negative stereotypes about mental health problems, creating a positive behavioral intention in the target audience	A reduction in stigma toward people with mental health problems was determined. People saw themselves as less stigmatizing compared to other people	The sample was opportunistic and there was no comparison group. The study was modest in size and did not include a follow-up period. The questionnaire used in the evaluation was not a standardized instrument. There was a lack of qualitative data to understand the changes
Reavley et al. (2018)	Australia	State civil servants (*N* = 608)	Public sector Government organizations	Randomized controlled clinical trial design	The MHFA (Mental Health First Aid) program was applied in e-learning or mixed modality (face-to-face and e-learning), incorporating a series of interactive and didactic resources. The intervention lasted 10 h, six online and four face-to-face	Look for changes in knowledge, stigmatizing attitudes, confidence to provide support, and intention to support a person with depression or post-traumatic stress disorder	The compared interventions had positive effects on the outcome variables. In turn, the mixed MHFA intervention was slightly more effective than MHFA e-learning in improving knowledge and stigmatizing attitudes	The larger than expected attrition and consequent lack of power to assess differences between the two modes of MHFA delivery. An additional limitation is the fact that intentions may not translate into actual behaviors
Shann et al. (2019)	Australia	Leaders of organizations (*N* = 196)	Sectors not reported Organizations from different sectors of activity	Field randomized controlled clinical trial design	This is a brief online workplace mental health intervention aimed at leaders. No information is presented on the duration of the intervention	It seeks to reduce the stigma related to depression in the leaders of an organization, as well as to identify key factors in the transfer of learning	Results revealed significant reductions in stigma toward behavioral and affective components of depression among leaders who completed the intervention. Attitudes and knowledge are insufficient to ensure the transfer of learning, with factors such as work environment, collective willingness, organizational capacity to solve problems, attitudes of others at work, and the broader political context playing an important role	Factors including the nature of the work environment, the collective readiness and capability of the organization to address these issues, the attitudes of others at work, and the broader political context affected the application of learning from the intervention. Another limitation is the specificity of the sample of workers (only leaders of organizations)
Svensson and Hansson (2014)	Sweden	Staff of social security offices, employment agencies, social services, schools, police departments, correctional treatment units, rescue services, and recreation centers (*N* = 561)	Public sector Public sector organizations, public administration	Randomized controlled clinical trial design, with two follow-up times	It measures the effectiveness of the MHFA (Mental Health First Aid) program. Intervention with a duration of 20 h, distributed in 2 days	It seeks to increase awareness of mental disorders and supportive behavior and improve attitudes toward these people	Both knowledge and confidence in providing help to someone in need improved. At the two-year follow-up, the improvements were sustained	The sample is not representative of the general public. The majorities of the participants had a high level of education and were women and generalizations must be made with caution. The attrition rate between base-line and six months follow-up was rather high
Szeto et al. (2019)	Canada	First responders (e.g., police, firefighters, paramedics, emergency services) (*N* = 4649)	Sectors not reported, although the public sector is predominant	Pre-experimental design (Pre- and post-test design)	With a 3-month follow-up in 5 groups of first responders in 16 workplaces. The Road to Mental Readiness for First Responders (R2MR), a resilience and anti-stigma program, was tested with meta-analytic methods, with a regular duration of 4 h or an extended period of 8 h	It seeks to decrease stigma toward mental disorders and increase resilience. The extended version also incorporates creating a supportive work environment and following up after stressful events	The intervention effectively reduced mental health stigma and increased resilience skills after program implementation in participants from the various work settings of the first responder groups	The study design was a pre-post test with a follow-up open trial. Despite the large pre-post sample, there was substantial attrition at the follow-up time point. Finally, the current study conducted follow-up assessment at a 3-month period; this may not be sufficient to assess its effects
Tynan et al. (2018)	Australia	Coal mining workers (*N* = 1275) and their supervisors (*N* = 117)	Private sector Coal mining industrial sector	Pre-experimental design (Pre- and post-test design)	This multi-component program includes the MATES intervention (peer-based mental health and suicide prevention program) and training aimed at supervisors. The duration of each specific component of the program is reported from 1 h to 2 days	It seeks to evaluate the feasibility, acceptability, and effectiveness of a peer-based program focused on mental health and suicide prevention, specifically on its identification and promotion of supportive behaviors	Both workers and supervisors were more confident that they could identify a co-worker with mental health problems, help a co-worker, family member, or themselves identify where to get support. They were also more willing to talk to a co-worker about mental health	The design was pre-post and therefore lack a control group. The process evaluation measured participants' perceptions and attitudes, but did not determine whether this translates into changes in their behavior

### Scope of application

This category groups the target population or participants and the private or public sector in which the intervention is applied. It also refers to the work and/or organizational setting in which it is implemented. The setting is the sector of activity or organizational environment (in terms of processes or structures) where the intervention occurs. Finally, the category includes the country where the intervention takes place.

A review of the shared characteristics of this category reveals at least three attributes or dimensions that cut across these studies. On the one hand, there is a greater application of these interventions in the public sector compared to the private sector or the application in both sectors. In this line, of the 21 studies in which the sector to which the organizations belong is identified, 52% (*N* = 11) correspond to the public sector, while 29% (*N* = 6) to the private sector and 19% (*N* = 4) to studies applied in both. The participants in the interventions are diverse in terms of occupations and the workforce to which they belong. This ranges from workers performing operational or functional tasks in various sectors of activity to people in managerial or supervisory roles. Detailing these data in descending order, of the 25 studies analyzed, 20% (*N* = 5) considered workers in managerial or supervisory positions, 20% (*N* = 5) corresponded to health professionals, another 16% (*N* = 4) were identified broadly as workers in different organizations, and 12% (*N* = 3) were identified as workers in various organizations, 12% (*N* = 3) were civil servants, 8% (*N* = 2) were military, another 8% (*N* = 2) included workers in the industrial manufacturing sector. In smaller proportions, studies were found with police officers (4%, *N* = 1), firefighters (4%, *N* = 1), first responders (4%, *N* = 1), and school teachers (4%, *N* = 1).

Concerning work settings, there is a greater presence of focused interventions in public government (16%, *N* = 4), health (16%, *N* = 4), industrial manufacturing (12%, *N* = 3), business in different sectors of activity (12%, *N* = 3) and police and public protection services (12%, *N* = 3). This is followed by studies in education and telecommunications (8%, *N* = 2) and military (8%, *N* = 2). The lowest presence of studies is in the agriculture sector (4%, *N* = 1), construction sector (4%, *N* = 1), non-governmental organizations (4%, *N* = 1), and unidentified organizational settings (4%, *N* = 1). Finally, Anglo-Saxon countries show greater development and implementation of these interventions, especially Canada (32%, *N* = 8), Australia (20%, *N* = 5), and the United Kingdom (20%, *N* = 5). Other developed countries such as Japan (8%, *N* = 2), Germany (4%, *N* = 1), Denmark (4%, *N* = 1), Sweden (4%, *N* = 1), and developing countries such as South Africa (4%, N = 1) also have actions along these lines. It is noteworthy that, of the Anglo-Saxon countries, the United States of America has the lowest number of studies (4%, *N* = 1).

### Design proposal and intervention modality

The category refers to the type of methodological design, the modality or type of intervention most used, as well as the duration and frequency of the intervention.

Regarding methodological design, 52% of the studies analyzed (*N* = 13) are randomized controlled clinical trials. They are followed by studies of pre-experimental design (pre-post-test in one group) (40%, *N* = 10) and quasi-experimental design (one experimental and one control group, non-randomized, with pretest and posttest evaluation) (8%, *N* = 2). Another relevant aspect is that most of them are training programs that include a wide diversity of characteristics, which can be systematized into some common categories: (a) They are interventions that include mental health literacy, and their format is usually standardized and manualized; (b) The interventions encourage the recognition of mental health risk behaviors, which in some cases takes the form of job induction; c) The importance of taking responsibility for mental health problems and possible discrimination or stigma is emphasized; d) Direct contact strategies are used to a lesser extent (such as narratives of service users), experiential group learning, peer support, didactic teaching approaches and the use of interactive resources; e) In line with the above, there is a growing trend in the incorporation of new technologies in the training actions implemented, whether in e-learning format, training based on digital games, among other innovations.

There is also a marked predominance of the intervention known as MHFA (Mental Health First Aid) as part of the change initiatives implemented, which is present in 43.4% of the studies analyzed (*N* = 10). This intervention consists of a protocolized international training program based on standardized mental health literacy, with a knowledge and skills training modality that fosters recognizing mental health risk behaviors in others.

Finally, it was noted that stigma reduction training in the different studies ranged from two hours to multiple sessions that may extend over several weeks.

### Objective of the intervention

Describes the goal that the intervention is intended to achieve and therefore involves establishing the focus(s) of the intervention in terms of the type of disorder and expected change (e.g., outcome measures such as attitudinal change, behavioral change, etc.).

At least two common core aspects characterize this category. First, it focuses on different disorders towards which to reduce stigma: a) Interventions with a focus on general mental health, these are the most numerous and reach 64% of the studies analyzed (*N* = 16); b) Interventions directed toward depressive and anxiety disorders, with 28% of the studies (*N* = 7); c) Interventions with a focus on post-traumatic stress disorder and stress, with 8% (*N* = 2). Secondly, although all the studies analyzed aim to reduce stigma towards mental health problems in work contexts, the specific objectives, and expected changes are mixed in the studies, making it difficult to classify them. In general terms, the researches aim to: a) Increase mental health literacy, either in general or in specific disorders, increasing the level of knowledge in this regard; b) Increase positive attitudes and decrease prejudices and stigmatizing attitudes; c) Increase orientation and support behaviors towards those who present mental health problems (positive behavioral intention, confidence, and self-efficacy); d) Increase communication skills and recognition of mental health problems; e) Increase help-seeking behaviors in those who have mental health problems. In addition, some studies point out a general objective to promote mental health at work to increase the well-being of workers.

### Impact of the intervention

Finally, this category refers to the evaluation of the impact of the intervention in terms of the results obtained through indicators that assess its effectiveness in reducing stigma. It also includes an assessment of their potential limitations.

In this category, the research results were classified into five subcategories: 1) A total of 20 studies (80%) show an increase in positive attitudes and a reduction in stigmatizing attitudes (prejudice) towards mental health problems, in some cases general and others specific. Only two reports mixed results, i.e., a combination of expected and unexpected changes in the variables evaluated; 2) 69.56% (*N* = 16) indicate that participants increased their knowledge in mental health (in general and in particular disorders); 3) 10 studies (43. 47%) report changes in confidence and self-efficacy in actions to help people with mental health problems; 4) 36% (*N* = 9) showed increased help-seeking behaviors, as well as mental health promotion, of which only one reports unexpected changes, in one of the outcome measures used; 5) Only two investigations (8.69%) report changes in intention and attitude to support, and support-seeking, one in positive terms and another with mixed results.

Two of the 25 studies analyzed report changes maintained at six months (changes in knowledge, reduction of stigma, and increase in self-confidence in helping) and two years (improvement in knowledge and self-confidence in helping), respectively. From this, it can be deduced that the changes maintained are more attitudinal and knowledge nature but not of a behavioral nature.

In terms of limitations, the main ones are the study design in terms of the non-inclusion of the control group in the case of pre-experimental designs (pre-post-test in one group) (40% of the studies analyzed, *N* = 10) and the short longitudinal nature and lack of control in the case of quasi-experimental designs (one experimental and one control group, non-randomized, with pretest and posttest evaluation) (8%, *N* = 2), this is not the case for studies based on the randomized controlled clinical trials design (52%, *N* = 13), which is more like a strength. Other limitations identified were relatively small sample size (20%, *N* = 5), the studies was restricted to one type of organization (40%, *N* = 10), the studies attrition (24%; *N* = 6), evaluation using self-rated questionnaires (32%; *N* = 8), the short-term follow up period (20%; *N* = 5), the fact that intentions may not translate into actual behaviors (8%; *N* = 2), ability to identify the use and impact of training (8%; *N* = 2), lack of qualitative data to understand the changes (8%; *N* = 2), and participants might have been presensitized in public stigma reduction efforts (4%; *N* = 1).

## Conclusion

This is one of the few reviews on stigma in the workplace. The objective was to identify and describe the main characteristics of interventions to reduce mental health stigma in the workplace.

It highlights the scarcity of interventions aimed at these purposes; only 25 investigations were included. This result reaffirms the need for studies in this line of work. Organizational context is an environment where interventions for stigma reduction can be successful considering its nature, duration, intensity, and contextual specificity, among other factors, pointed out by different authors (Hanisch et al., 2016; Krupa et al., 2009; Szeto & Dobson, 2010).

Regarding the characteristics of the interventions, the military, government, healthcare, and manufacturing contexts, with greater representation of the public sector, are where a greater number of these programs have been developed. This shows the concern and investment of resources in improving the mental health of public servants, as well as the need to include these interventions in other workplaces beyond public services.

As has been found in several reviews on stigma, not only in the workplace, Anglo-Saxon and developed countries are the ones leading interventions to reduce it (Mehta et al., 2015), while research is needed in developing countries because considering the cultural aspects of the phenomenon (Mascayano et al., 2016; Yang et al., 2007). Stigma is a social phenomenon and cultural aspects shape it. For example, in Latin American countries, stigma is related to gender roles; women who cannot exercise the role of caregiver and men the role of provider, linked to a patriarchal culture, tend to be more stigmatized (Mascayano et al., 2016; Yang et al., 2014). In general, stigma reduction programs are developed in Western countries, as was seen in this review, which cannot always be introduced in places with other values and material resources (Mascayano et al., 2020). The study by Jorm et al. (2010) was the only one conducted in a middle-income country such as South Africa. In this study, the content of the Mental Health First Aid training course was adapted to the particular context, detecting the most frequent mental health problems in young people in schools, establishing contextualized approach plans and using the Department of Education and local children's services as a strategic partner. It is, therefore necessary to conduct research in developing countries and determine which components need to be modified to take into account the context and which can be used without major changes.

Intervention formats are diverse in the number of hours and strategies used. Regarding duration, interventions to reduce stigma in other populations (university students, health professionals, and patients) have found the same, i.e., the extension is variable and the minimum amount of time required to achieve effectiveness is unclear (Gronholm et al., 2017; Morgan et al., 2018). Therefore, it is important to make progress in determining the number of hours that an intervention should take to obtain positive results. This time is related to the strategies used and their modality of implementation. In terms of strategies, the Mental Health First Aid program, which has as one of its focuses on the reduction of stigma, but focuses on providing support to co-workers who have a mental disorder, stands out. From this perspective, programs to reduce stigma in the workplace focus primarily on improving workers' mental health rather than on other components of the organizational system, such as users. In the health care setting, interventions to reduce stigma have among their main target populations workers; however, their focus is on how they care for consultants (Henderson et al., 2014; Knaak & Patten, 2016). Interestingly, these two types of interventions, directed towards staff and users, establish a common ground to enhance, in workplaces where people with mental health problems are frequently attended, an intervention that serves both purposes, i.e., to reduce stigma towards both workers and users.

As workplace interventions are oriented toward workers, they have an important educational component that facilitates the participant to recognize mental health problems to seek and provide help. Although interventions to reduce stigma in other groups also use mental health literacy as a strategy (Bingham & O'Brien, 2018; Stuart et al., 2012), they often use contact because of the positive effects it has on reducing stigma (Henderson & Gronholm, 2018; Mascayano et al., 2015; Thornicroft et al., 2016). Their lesser use in workplace interventions, on the one hand, reaffirms that stigma reduction is not the central focus of interventions and, on the other hand, shows that other strategies, such as skills development, can be effective. Programs in the work context include skills training to recognize and support co-workers with mental health problems. The emphasis given to skills development could be an important contribution to programs to reduce stigma among healthcare workers who, as they are user-facing, primarily use education and contact as strategies (Fokuo et al., 2017; Knaak & Patten, 2016).

Most interventions focus on a specific disorder rather than mental disorders. Targeting allows for the delivery of information atingent to a condition, which increases the effectiveness of education (Stuart et al., 2012), and in this particular case, increases strategies for providing support to a co-worker.

The design of the interventions includes pre- and quasi-experimental, and most occupy a randomized clinical trial (RCT) design. However, pre- and quasi-experimental designs together are almost as numerous as experimental designs. This is relevant as an RCT maximizes the control of variables that could interfere with the results (Gronholm et al., 2017); therefore, it is important to continue advancing in the use of more rigorous methodological designs such as this one.

The main outcome measures focus on increasing mental health knowledge and confidence in providing help, changing attitudes toward help-seeking and people with mental disorders, and proxy measures of behavioral change such as decreasing social distance. In general terms, the interventions are effective, i.e., they generate significant changes in these variables. However, this area of stigma reduction, like others, lacks measures of direct behavioral change (Corrigan & Shapiro, 2010; Stuart et al., 2012), which is a limitation because although knowledge and attitudes contribute to determining behaviors, the relationship is not direct, so their modification does not imply a real behavioral change (Corrigan & Shapiro, 2010; Stuart et al., 2012). Perhaps, one of the main challenges of research on this topic is to incorporate behavioral change measures, considering the practical difficulties that exist for their implementation in natural contexts, such as work. On the other hand, outcome variables are individual. For some years, it has been considered that organizational aspects such as culture and structure play a role in the formation of attitudes and behaviors, so it is recommended to consider them when intervening (Cook et al., 2014; Henderson et al., 2014), especially when considering stigma from an ecological model.

Including organizational variables is a challenge for workplace interventions because although, in general, these programs are favorable and show that there is an improvement in knowledge, attitudes, and supportive behaviors towards people with a psychiatric diagnosis (Hanish et al., 2016), so far the workplace is only considered as a setting, not including a decided organizational look at it, being an important future challenge (Thomson & Grandy, 2018). Some of the studies analyzed, to give sustainability to changes in follow-up, emphasize the critical character of different factors such as the nature of the work environment, the collective and team disposition towards mental health, the organization's capacity to address these problems, the use of contextually relevant examples, support from all levels of the organization, among others (e.g., Moll et al., 2018a, 2018b; Shann et al., 2019).

It appears that the effect of the interventions is sustained over time. However, this result is very incipient due to the scarcity of studies that contemplate follow-up, so research is needed to determine the medium- and long-term effects of the programs (Gronholm et al., 2017; Thornicroft et al., 2016). On the other hand, in addition to the design limitations of the interventions highlighted in the results (particularly in pre-experimental and quasi-experimental designs), there are also others that cut across the different studies, notably the relatively small sample size, the restricted type of organization considered, the studies attrition, the evaluation using self-rated questionnaires and the short-term follow up period, among others.

In summary, it is possible to point out that the main challenges and recommendations for research on interventions to reduce mental health stigma in work settings are the following: the scarcity of research on cultural aspects specific to developing and undeveloped countries, which needs to be strengthened in order to identify key factors for replicability and effectiveness; the importance of determining the duration of an intervention to achieve positive results; the need for interventions that serve both to reduce stigma towards those who work and towards the users/clients of the organization; the emphasis on skills development in these interventions to reduce stigma towards those who work and towards the users/clients of the organization; the emphasis on skills development could be an important contribution to programmes to reduce stigma with health workers in direct contact with users; it is recommended that further progress be made in the use of more rigorous methodological designs such as randomized controlled clinical trial design; it is essential to incorporate measures of behavioral change to estimate the impact of interventions, as well as variables of an organizational nature that provide the context and psychosocial support for such changes; more research is also needed on the medium and long-term effects of programmes focused on the workplace.

One of the limitations of this review was that only articles in Spanish and English were considered, so any other contributions in the area were not included. On the other hand, there is a disparity of methodological designs, making it difficult to compare the results; also, not all studies present indicators of the changes achieved, such as effect size. For this reason, it is important that future research advances using rigorous methodological designs that show the magnitude of the results achieved.

This review established four domains of analysis of interventions to reduce stigma in the workplace. Based on these criteria, it was possible to highlight common attributes among the different interventions, which facilitated their characterization and understanding, thus contributing to the advancement of this field of knowledge.

The workplace is a privileged space to intervene in the reduction of stigma because it has a captive population with which it is possible to work from an ecological model in programs that consider intervention strategies by levels, which would favor individual changes likely to apply in the context where they were generated, and changes in the organization to make it more inclusive.

## Data Availability

The data that support the findings of this review are available from the corresponding author upon reasonable request.

## Abbreviations

MHFA: Mental Health First Aid
RCT: Randomized clinical trial
